# *Alhagi maurorum* extract modulates quorum sensing genes and biofilm formation in *Proteus mirabilis*

**DOI:** 10.1038/s41598-022-18362-x

**Published:** 2022-08-17

**Authors:** Arezoo Mirzaei, Bahram Nasr Esfahani, Mustafa Ghanadian, Sharareh Moghim

**Affiliations:** 1 Department of Bacteriology and Virology, School of Medicine, Isfahan University of Medical Sciences, Isfahan, Iran; 2 Department of Pharmacognosy, School of Pharmacy, Isfahan University of Medical Sciences, Isfahan, Iran

**Keywords:** Antimicrobials, Bacteriology, Biofilms

## Abstract

*Proteus mirabilis* (*P. mirabilis)* is a frequent cause of catheter-associated urinary tract infections. This study aims to investigate the anti-infective effect of *Alhagi maurorum* extract (AME), the traditional medicinal plant in the middle east, on the biofilm-forming *P. mirabilis* isolates. Hydroalcoholic extract and oil of *A. maurorum* were characterized by HPLC and GC–MS. The antiproliferative, anti-biofilm, and bactericidal activity of AME at various concentrations were assessed by turbidity, crystal violet binding, and agar well diffusion assays, respectively. The AME’s effect on adhesion and quorum sensing (QS) were investigated by in vitro adhesion assay on cell culture and agar overlay assay using *Janthinobacterium lividum* (ATCC 12472) as a biosensor strain. In addition, the expression level of selected genes involved in QS and biofilm regulation were determined by quantitative Real-Time PCR. Furthermore, the bladder phantom model was created to evaluate the assays and investigate the catheter’s calcium deposition. The most effective chemical compounds found in AME were tamarixetin, quercetin, and trans-anethole. Although AME did not inhibit swarming motility, it reduced biofilm production and exerted a concentration-dependent anti-adhesive and anti-QS activity against *P. mirabilis*. AME also downregulated the expression level of selected genes involved in biofilm formation and QS. This study showed that AME as a natural compound reduced biofilm formation of *P. mirabilis* by targeting virulence factor genes, quorum sensing, and other strategies that include preventing the adhesion of *P. mirabilis* to the cells. The results suggest that *A. maurorum* extract might have the potential to be considered for preventing UTIs caused by *P. mirabilis*.

## Introduction

Urinary tract infections (UTIs) are one of the most prevalent nosocomial infections. Most UTIs are caused by Enterobacteriaceae. *E. coli* and *P. mirabilis* are the most common causal bacteria in the family, accounting for about 90% of complex UTIs^1^. The increasing use of antibiotics to treat UTIs has led to the development of multi-drug resistant (MDR) bacteria^2^. *P. mirabilis* UTIs, particularly in the patients undergoing long-term catheterization, cause the formation of urinary stones (urolithiasis) and long-term catheterized infections^3^. Among many virulence factors used by *P. mirabilis* Catheter-Associated Urinary Tract Infections (CAUTIs), some virulence factors were linked to their ability to form biofilms, such as swarming motility, fimbriae, urease production, capsule polysaccharide, and efflux pumps^4^. The ability to form strong biofilm is one of the major virulence factors in *P. mirabilis* causing UTIs. The biofilm formed by *P. mirabilis* has a critical role in UTIs and makes MDRs and recurrent infections to current antibiotics. Biofilm formation enhances the resistance to antibiotics as well as the host immune system^5^. In the recent decade, efforts to develop new antibiotics have reduced, while drug-resistant strains have become challenging^6^. Therefore, new alternative strategies are needed to eradicate biofilm-forming pathogens.

Plant essential oils and extracts are considered a rich source of a wide range of bioactive compounds known as phytochemicals^7^. The possible antibiofilm activity of phytochemicals has made interest, particularly in applying the plants as alternatives to treat infectious diseases^8^. Phytochemicals could inhibit bacterial adherence to the surfaces, quorum sensing (QS), urease activity, and adhesion to the exopolysaccharide matrix^4^. The *Alhagi maurorum (A. maurorum)* is a traditional medicinal plant (known as camelthorn or manna plant) and is found in the middle east, including Iran^9^. Many studies showed the medicinal properties and frequent use of *A. maurorum* in rheumatic pains, bilharzias, liver disorders, and gastrointestinal discomfort disease treatment. Furthermore, *A. maurorum* has a potential effect on treating UTI_s_ and acts as a powerful diuretic and antilithiastic^10^. Although the possible mechanisms of some phytochemicals assessed for *P. mirabilis* were studied^4^, the anti-biofilm activity and the molecular mechanisms caused by *A. maurorum* extract (AME) are unclear.

This study aimed to evaluate the effect of *A. maurorum* extract in biofilm degradation and QS genes expression of *P. mirabilis* isolated from the urinary catheters.

## Results

### Gas chromatography-mass spectrometry (GC–MS)

**Figure 1:**
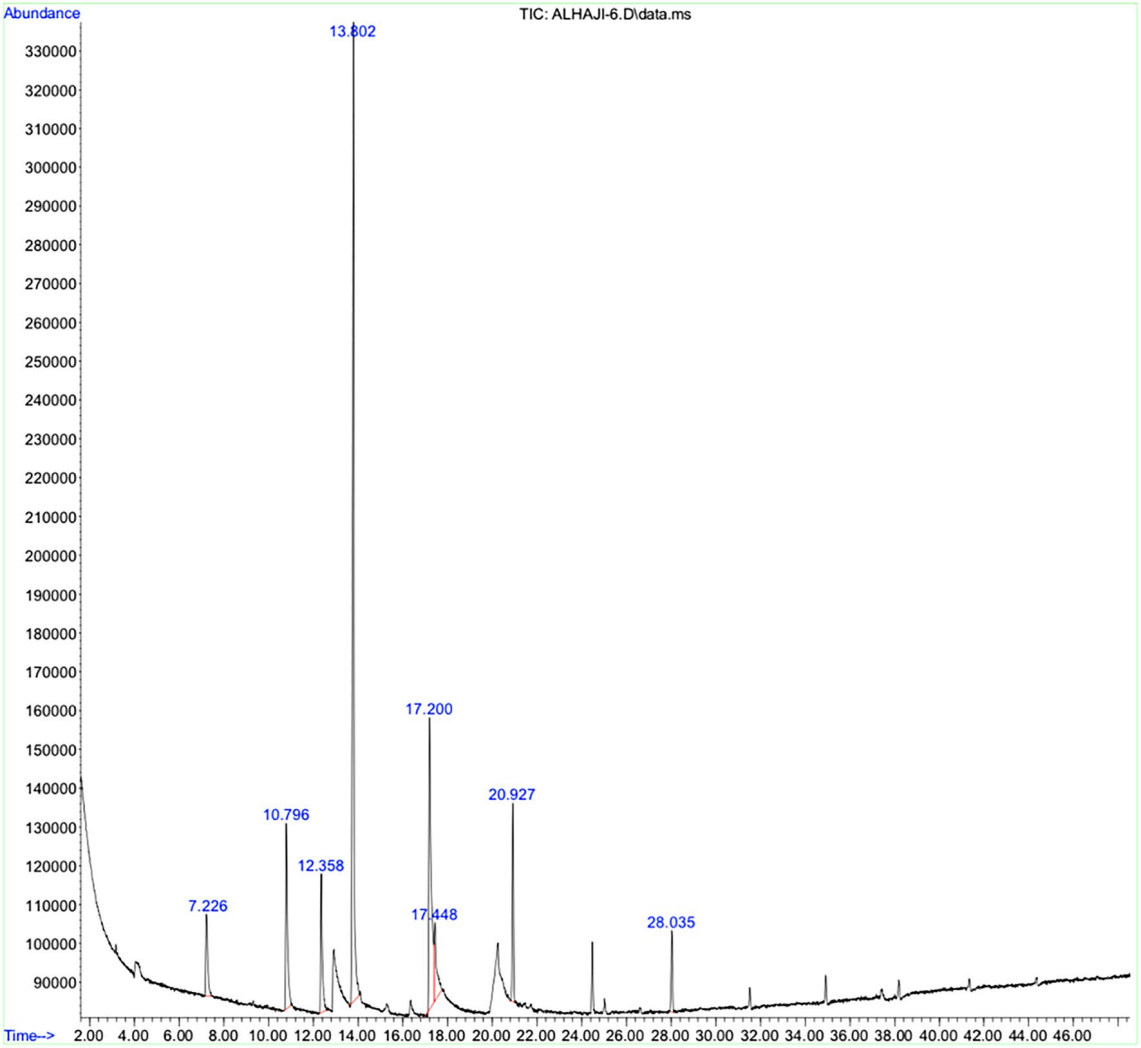
Gas chromatography spectra of biologically active compounds of *Alhagi marurum*.

The analytic results of the bioactive compounds present in the aqueous extract of *A. maurorum* essence by GC–MS showed the presence of 8 bioactive phytochemical compounds. The highest percentage content of the compounds are as follows: Trans-Anethole (41.96%), 2-Propanone, 1–4-methoxyphenyl (20.46%), Benzene,1-methoxy-4-(2-propenyl) (9.54%). Other active compounds with their peak number, concentration (peak area%), and retention time (RT) are presented in (Table 1, Fig. 1).

**Table 1:** Descriptors of the volatile components detected by GC–MS. Significant values are in bold.

Peak	Compound	Retention time (min)	Formula	Peak area
1	L-FENCHONE	7.226		4.8404
2	Benzene, 1-methoxy-4-(2-propenyl)	10.796		9.5458
3	2-Cyclohexen-1-one, 2-methyl-5-(1-methylethenyl)	12.358		6.7363
4	**TRANS-ANETHOLE**	**13.802**		**41.9664**
5	2-Propanone, 1-(4-methoxyphenyl)-	17.2		20.6406
6	1-Bromomethyl-4-methoxybenzene	17.448		5.7836
7	Tetradecane	20.927		7.1722
8	Hexadecane	28.035		3.3149

### High-performance liquid chromatography (HPLC)

**Figure 2:**
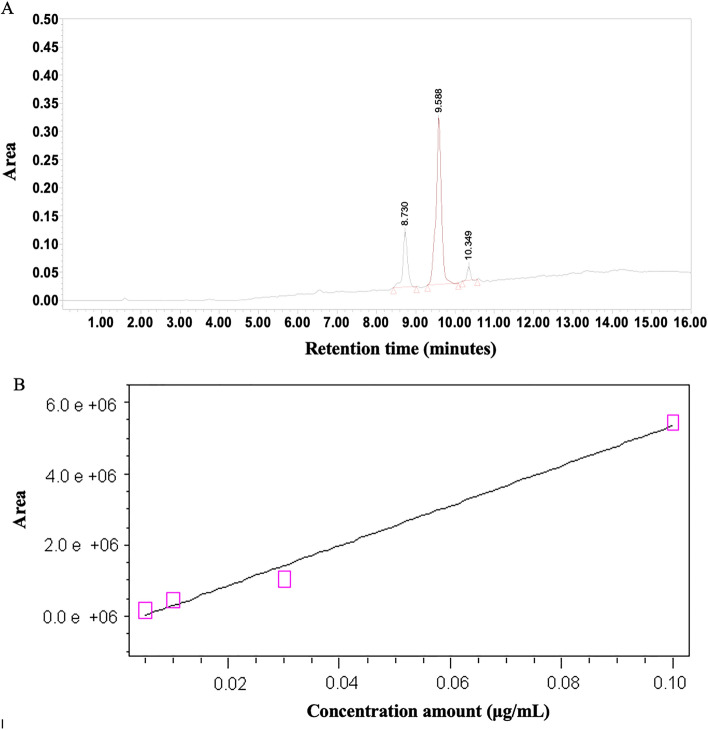
HPLC results of crude extract of *Alhagi maurorum*. (**A**) Result of crude extract, (**B**) Calibration curve of quercetin using HPLC analysis method.

The dried extract was standardized using quercetin and tamarixetin as the bioactive marker for the standardization of the extract. Quercetin and tamarixetin peaks of extract appeared at a retention time of 8.730, 9.588 min, respectively. Using a calibration curve, the extract was standardized to contain 19 μg/100 mg of quercetin and 55 μg/100 mg of tamarixetin (Fig. 2).

### Microbial isolation and identification

Of the 40 *P. mirabilis* isolates, 34 (82.5%) were MDR, twelve isolates (30%) had strong biofilm ability (OD590nm ≥ 2.5), twenty of them (50%) were moderate biofilm producer (1.5 ≤ OD 590 nm < 2.5), and eight (20%) of them had weak biofilm (0.7 ≤ OD590 nm < 1.5).

### Determination of antimicrobial activity, growth inhibition analysis, and minimum inhibitory concentration (MIC) of AME and the bioactive compounds of AME (quercetin, tamarixetin, and trans-anethole)

**Figure 3:**
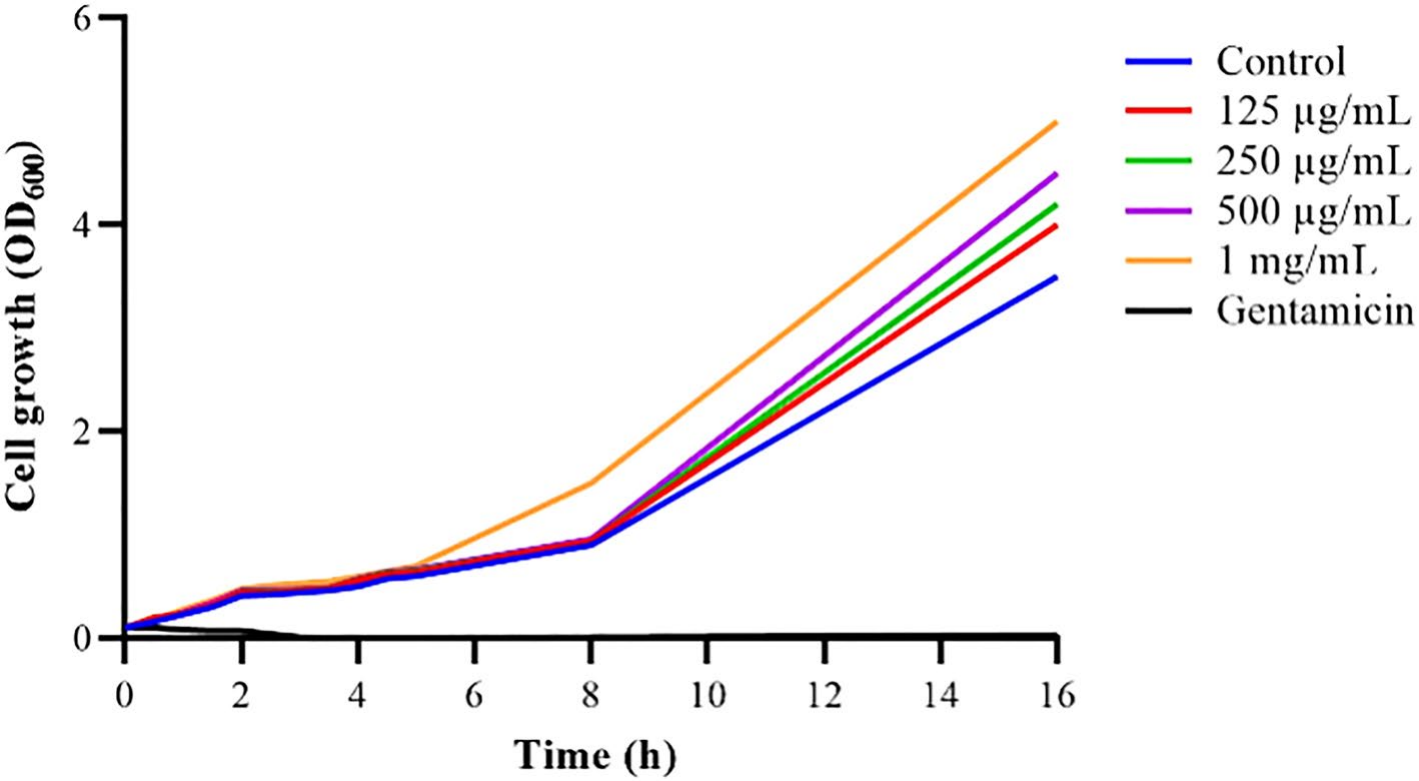
The effect of *Alhagi maurorum* extract at different concentrations (1000–125 µg/ml) on the growth of *P. mirabilis* in TSB media (solid line) or supplemented with the optimal value of *Alhagi maurorum* aqueous extract (dashed line). The data represent the absorbance of the mean ± SD values of experiments performed in triplicates.

The bactericidal assay of AME was evaluated by the agar well diffusion method. None of the AME concentrations (125–1000 μg/mL) showed a bacteriocidic effect on the *P. mirabilis* over an 18 h incubation time. The bacterial cultures treated with AME at different concentrations did not show any significant inhibitory effect on the growth of *P. mirabilis* compared with the control (Fig. 3). The AME didn’t show any inhibitory effect on *P. mirabilis,* but MIC_50_ of trans-anethole was at 1 mg/mL. The inhibitory effect of tamarixetin and quercetin was 23% and 12% at 500 μg/mL compared to the control.

### Motility inhibition assay

The swarming motility of *P. mirabilis* ATCC7002 was clearly visible in control and in AME and tamarixetin supplements at concentrations from 0.125 to 1 mg/ml. The bacteria exhibited a total swarming diameter of 20 mm in the treated and untreated experiments (*P* > 0.05). The concentration of quercetin and trans-anethole with 1 and 2 mg/mL, respectively, showed swarming inhibition at 25% and 50% of swarming plates.

### Qualitative QS inhibition assay

**Figure 4:**
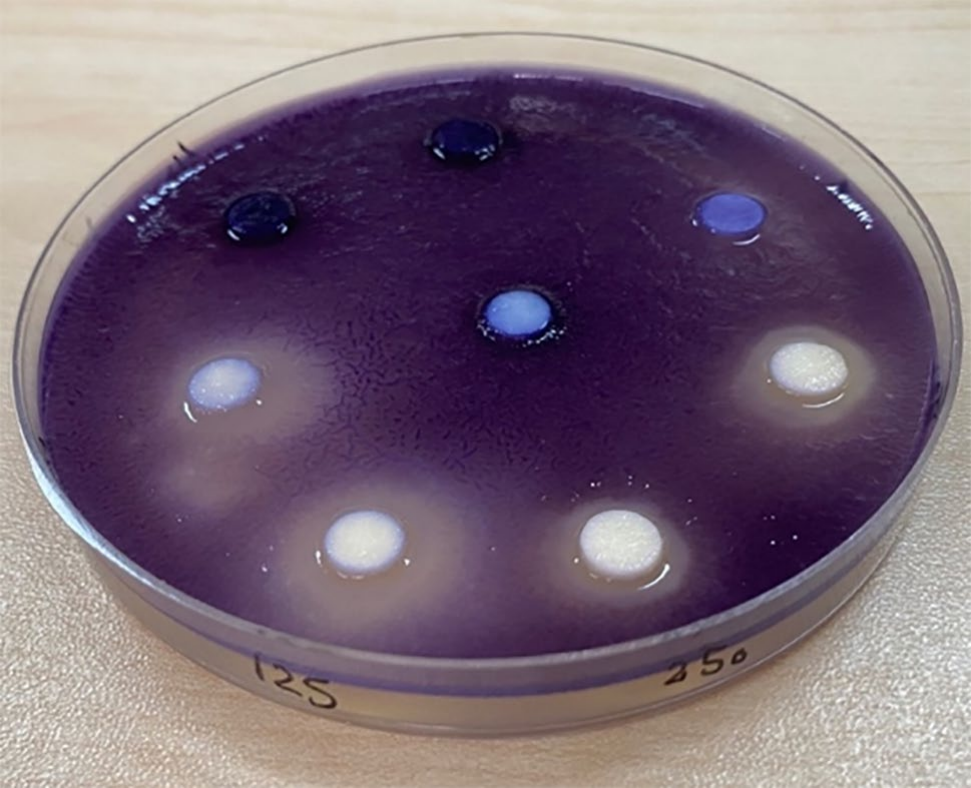
The qualitative anti-QS activity of different concentrations of AME.

*J. lividum* synthesizes the violet pigment violacein as a result of QS. Loss of purple pigmentation of *J. lividum* in the vicinity of the plant extracts indicated QS inhibition by the plant extract, which was seen in 62.5, 125, 250, and 500 μg/mL (Fig. 4).

### Quantitative anti-QS activity: violacein inhibition

**Figure 5:**
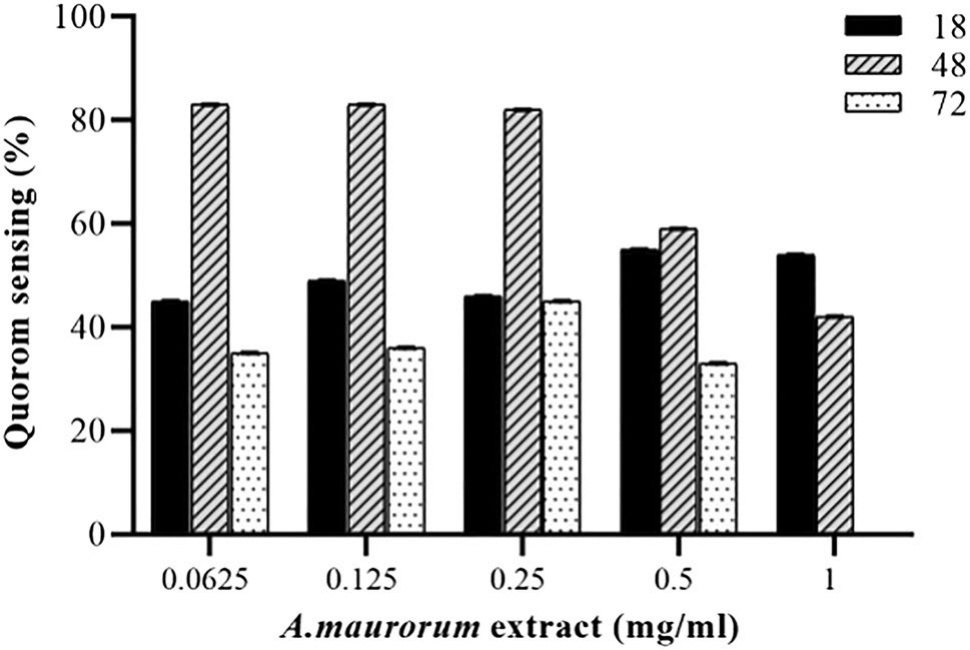
The percentage reduction of quantitative QS in different concentrations of AME and different times of exposure (18 h, 48 h, and 72 h).

The inhibitory effect of all *Alhagi* crude extract on the violacein pigment production at different concentrations (62.5–1000 μg/mL) was measured spectrophotometrically and quantified. The violacein pigment production decreased as the AME concentration increased (Fig. 5). This experiment was performed at different times (18, 48, and 72 h), and the optimum concentration of 125 μg/ml AME was considered for the following experiments.

### Eradication of biofilm

**Figure 6:**
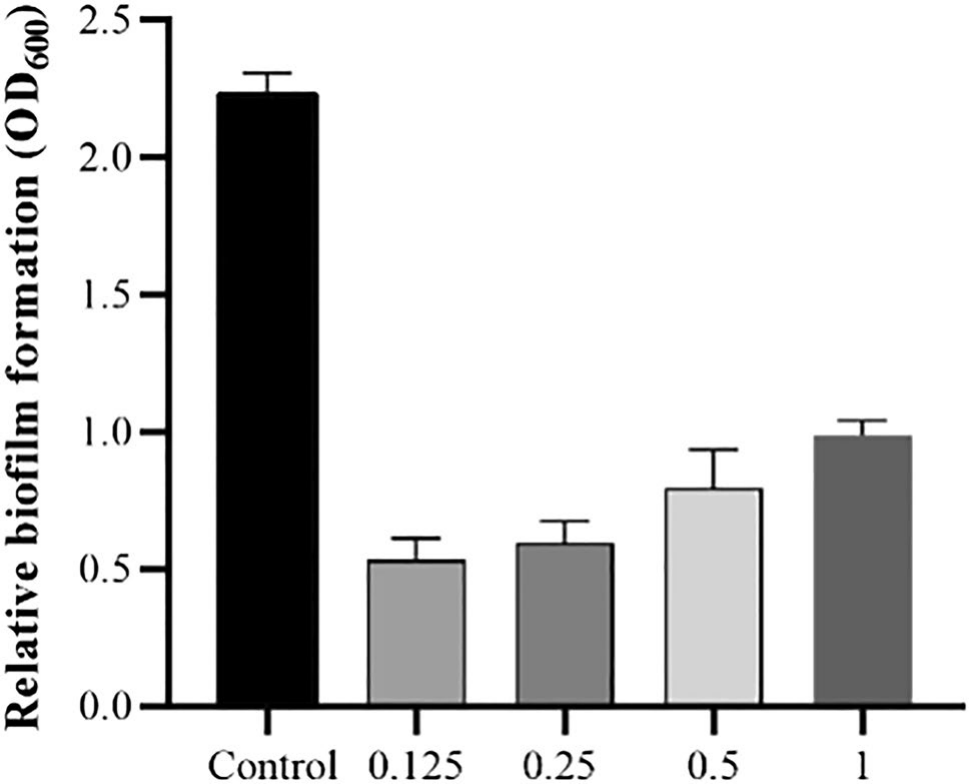
The effectiveness of AME with a different concentration in preventing biofilm formation of *P. mirabilis*.

As shown in Fig. 6, AME significantly inhibited biofilm formation. AME and its bioactive compound (quercetin, tamarixetin, and trans-anethole) inhibited the biofilm formation concentration-dependent. The bacteria were treated with AME at 125, 250, 500, and 1000 μg/mL; the biofilm inhibition rate was approximately 74%, 71%, 60%, and 54%, respectively. Quercetin had the most effective compound in AME with 80%, 95%, and 96% biofilm eradication at 125, 250, and 500 μg/mL. The second effective compound of AME was trans-anethole with 44%, 62% and 79% biofilm eradication at 500 μg/mL, 1 mg/mL, and 2 mg/mL. tamarixetin showed a lower reduction in biofilm with 45% and 49% at 62.5 and 125 μg/mL of concentration.

### Cell viability assay

**Figure 7:**
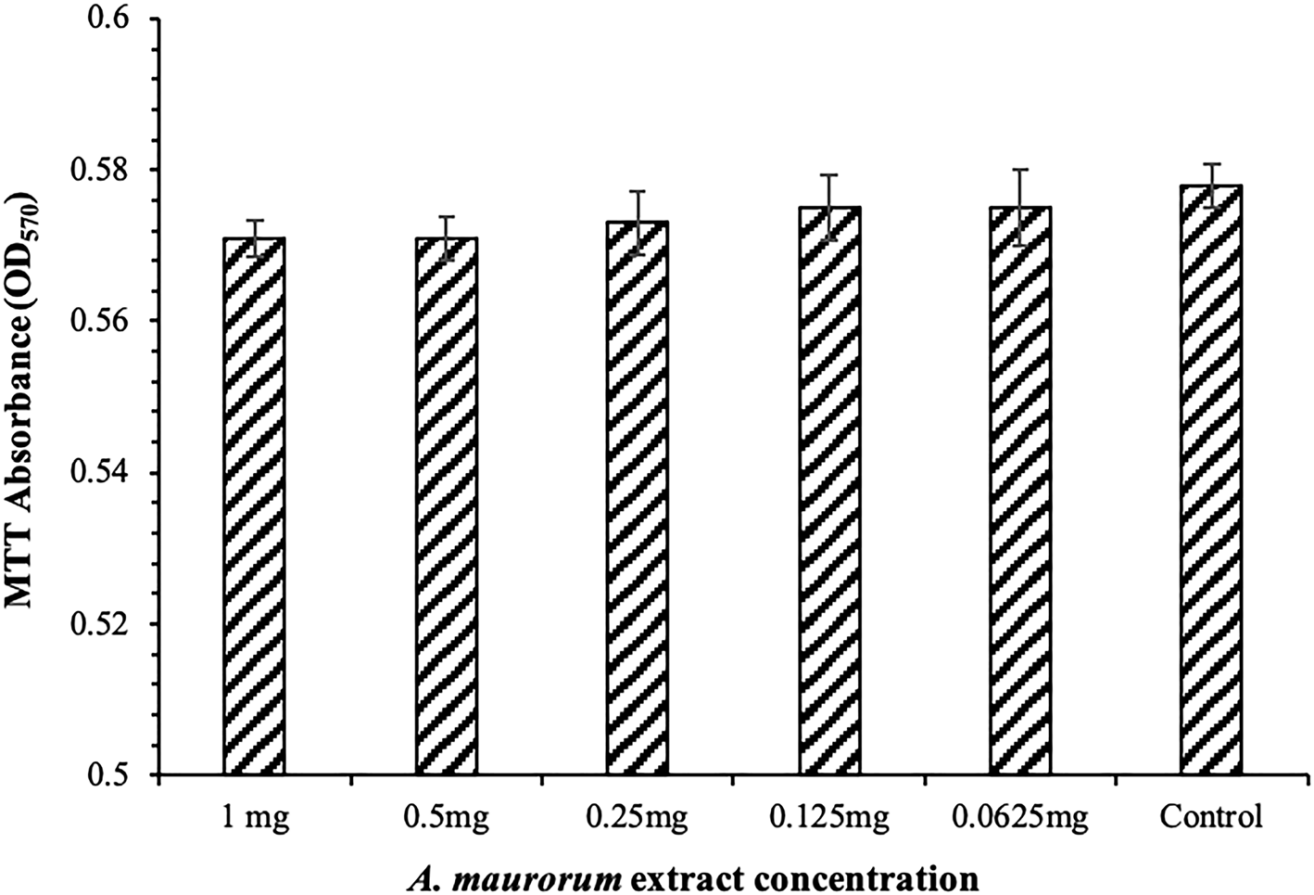
Survival assay with MTT. The absorbance of different concentrations of AME at OD570 nm.

The cytotoxicity of the AME was examined in HeLa cells. Cells treated with the AME at different concentrations (62.5–1000 μg/mL) survived as well as the control cells (*P* > 0.05), indicating that a high dose of AME did not affect HeLa survival (Fig. 7).

### Adhesion assay

**Figure 8:**
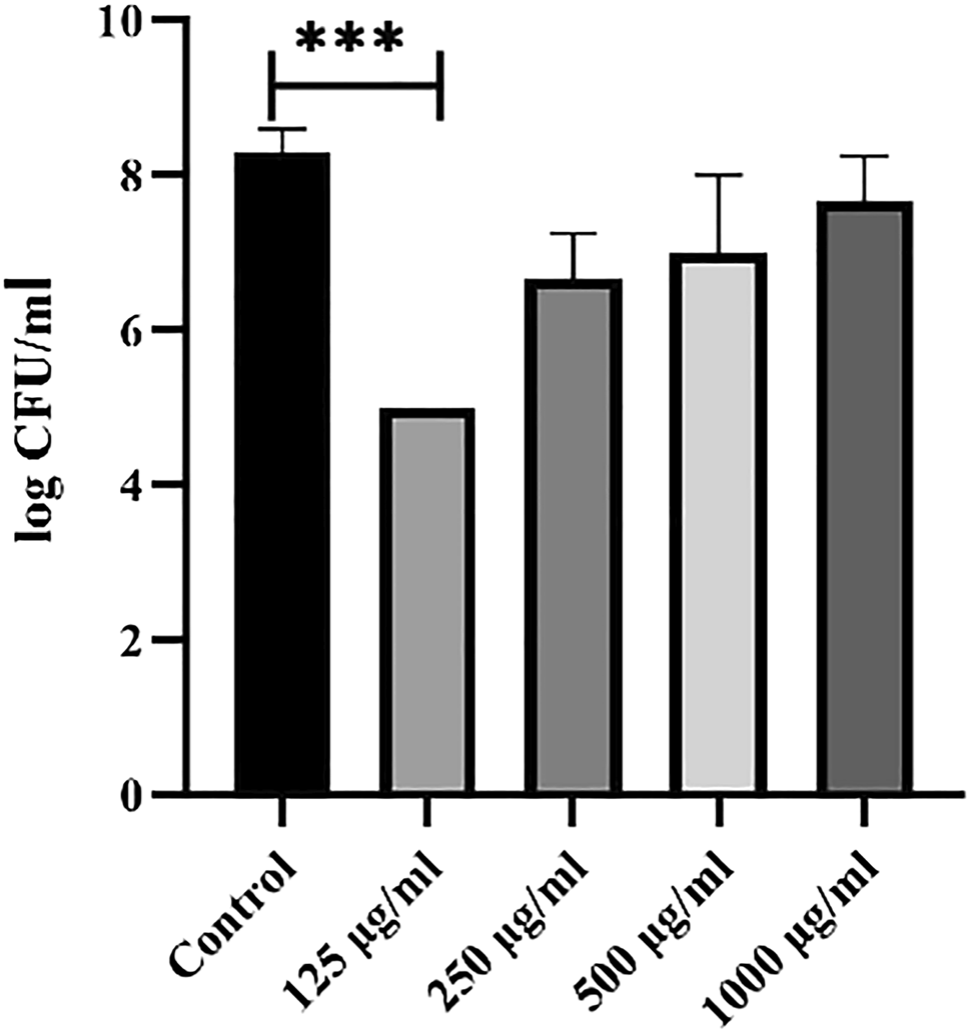
The effect of the different concentrations of AME on adhesion of *P. mirabilis* to HeLa cell.

The quantitative binding of *P. mirabilis* was investigated on the HeLa cell line by enumeration by plating on TSA. AME at different concentrations (125–1000 μg/mL) decreased the adherence of *P. mirabilis* to the HeLa cell line in a concentration-dependent manner. Results showed that at the concentration of 0.125 mg/ml of AME, *P. mirabilis* presents a 40% reduction in adhesion to HeLa cells (Fig. 8). Although, at the higher concentration of the extract (0.5 and 1 mg/ml), no significant reduction in the adhesion of *P. mirabilis* to HeLa cells was seen compared to the control (*P* > 0.05).

### Bladder phantom model

**Figure 9:**
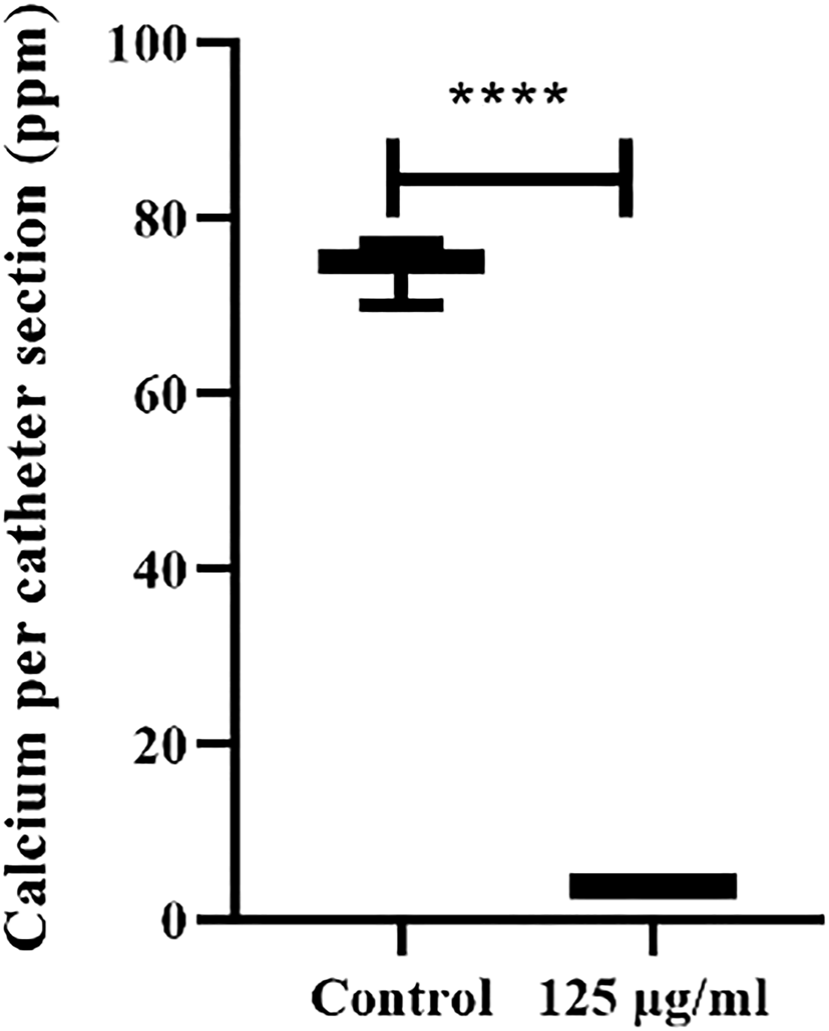
Impact of AME with an optimal concentration on crystalline biofilm formation on the catheter. ***P < 0.001.

To precisely evaluate the impact of AME on crystalline biofilm formation, models of late-stage infection were deactivated after 18 h, and calcium levels on catheter sections were quantified. As demonstrated in Fig. 9, AME significantly reduced the levels of encrustation at the concentration of 0.125 mg/ml. The urine pH was measured after treatment, and there was no significant difference in urine pH after treatment of extract in comparison with control (*P* > 0.05).

### Effect of AME on gene expression

**Figure 10:**
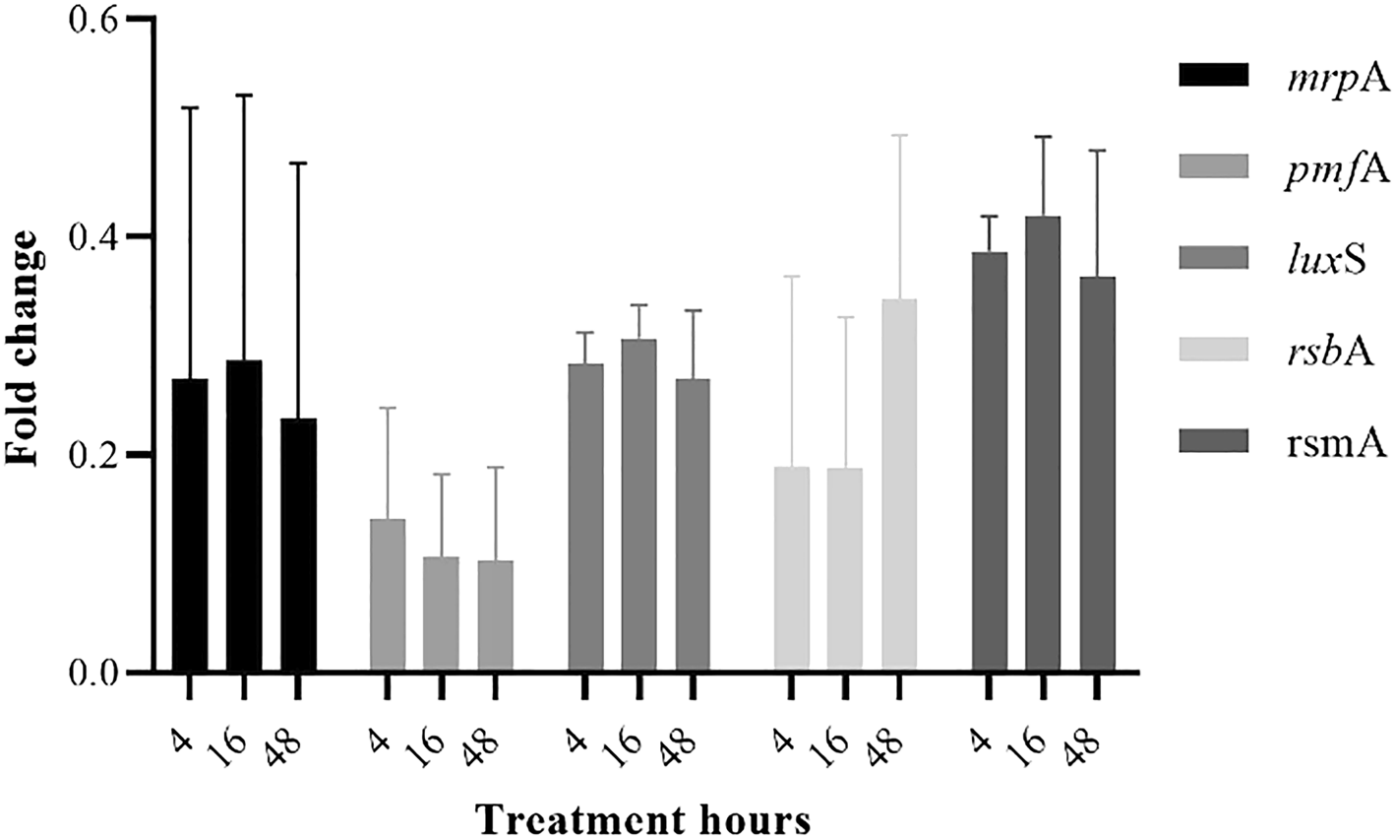
Relative expression and fold change according to the exposure time with *Alhagi* extract. A significant reduction was demonstrated especially in 16 h treatment.

We used a qRT-PCR assay to examine the effect of AME at an optimal concentration of 125 μg/ml on the adhesion and quorum sensing gene expression levels. Results showed that all of the *mrpA*, *pmfA*, *luxS*, *rsmA,* and *rsbA* genes were significantly downregulated, and their expression levels were reduced approximately by 2^−3.9^, 2^−5.6^, 2^−1.6^, 2^−4.5^, and 2^−1.4^-fold, respectively. Among examined different time intervals (4, 16, and 48 h), a significant reduction in the expression of these genes was seen after 16 h treatment (*P* < 0.05) (Fig. 10).

## Discussion

Antibiotic resistance in bacterial biofilms piqued researchers' interest in looking for additional anti-biofilm drugs and alternative therapeutics. Plants have long been thought to be a rich source of phytochemicals, which are bioactive compounds. Medicinal plants are a good substitute for commonly used antimicrobial drugs^11^. Among their various applications, phytochemicals have attracted particular interest to their antibiofilm activity, which was attributed to the inhibition of virulence factors, including microbial adherence, quorum sensing, urease activity, and exopolysaccharide matrix production^4^.

*A. maurorum* extract is composed of chemical compounds that show good antibacterial activity against different bacterial pathogens, including *Escherichia. coli*, *Staphylococcus. aureus, Bacillus. subtilis, Pseudomonas. aeruginosa* and *Salmonella. typhimurium*^12^. The phytochemical analysis of AME by GC–MS and HPLC revealed the presence of Trans-Anethole (p-methoxy propenyl benzene), tamarixetin, and quercetin. Trans-Anethole (tA), as a significant component of many essential oils, is an organic compound and a by-product of terpene synthesis^13^. Kwiatkowski et al. reported the significant antibacterial activity of tA on *S. aureus.* They showed that tA increased 2–3 times the inhibition zone of bacterial lawn and reduced 60%- 80% the biofilm formation of *S. aureus*^14,15^. In another study, Wieczyńska et al. showed the antimicrobial activities of some bio-based compounds, including eugenol, carvacrol, and trans-anethole (tA), against coliforms^16^. We examined the antimicrobial activity of *A. maurorum* extract, which showed the extract has neither a bactericidal nor bacteriostatic direct role against *P. mirabilis* isolates. These results were in line with those reported by Sosa^17^, who showed that *I. lutea* extract had no antibacterial and bactericidal activity against Uropathogenic *P. mirabilis* strains. In a study by Hadadi et al., the methanolic crude extract of *A. maurorum* had no antibacterial activity on some bacteria, including *B. subtilis*, *E. coli*, *Pseudomonas. syringae*, *Pseudomonas. viridiflava* and *Xanthomonas. campestris*^18^. Similarly, Ahmed et al. showed that the crude extract of *A. maurorum* was not active against *E. coli*^19^. Wang et al. study was assessed the inhibition effect of resveratrol compound on some virulence factor genes expression in *P. mirabilis,* and the results were similar to our findings^20^. We further investigated the swarming motility of *P. mirabilis* ATCC7002 phenotypically and observed clearly visible swarming in AME-treated bacteria and the control. Although, Aygul et al. showed that quercetin (as an active component of AME) inhibited the swarming motility of *P. mirabilis*. They supposed that the inhibitory effect of quercetin on *P. mirabilis* swarming was possibly in terms of regulating the expression level of polyamine enzymes which trigger the swarming differentiation or active pump proteins^21^.

The virulence factors such as swarming, biofilm formation, and the presence of an efflux pumping system are involved in the pathogenesis of *P. mirabilis* in UTIs^4^. We indicated that AME inhibits QS in a dose-dependent manner. The anti-QS activity of different plant extracts was investigated, and surprisingly, a wide range of natural products and traditional medicinal herbs showed significant anti-QS ability against Gram-positive and Gram-negative bacteria^22,23^. Another important virulence factor is biofilm formation. Several reports assessed the anti-biofilm activity of phytochemicals^24–26^. We evaluated the effect of AME on biofilm formation by crystal violet with the microtiter plate method. AME achieved up to 76% inhibition of biofilm formation at a concentration of 0.125 mg/mL. It is possible that the phytochemical compounds could inhibit bacterial growth in different pathways, including weakening the virulence of bacteria without showing bactericidal activity.

The inhibition of quorum sensing (QS) and initial attachment to cells may be related to the biofilm inhibition and decrease in adhesion to cells, for instance, the study which was done by Šimunović et al. showed that some of the natural extracts such as oregano, nettle, winter savory, roseroot, yarrow, and rosemary could reduce the motility and adhesion of *C. jejuni* via the modulation of LuxS (QS) system^27^. However, the molecular mechanisms of *Alhagi* hydroalcoholic crude extract have not been studied yet. To analyze the mechanism of the biofilm inhibition, the difference in expression levels of selected genes involved in biofilm formation and QS were evaluated by RT-qPCR. Gene expression analysis showed an altered pattern (2.6–20.7-fold) downregulation of the genes that affected the virulence properties of *P. mirabilis,* such as motility, biofilm formation, and QS activity. The data obtained from genotypic analysis confirmed the phenotypic results and showed AME could interact with biofilm and QS regulators in a dose- and time-dependent manner. Our data were in agreement with the data achieved from qualitative and quantitative QS inhibition assay performed by *J. lividum*. In agreement with our results, several reports illustrated the anti-QS,—biofilm formation, and -*lux*S expression of natural compounds^28–30^. Two existing genes, *rsmA* and *rsbA* in *P. mirabilis,* regulate swarming and virulence factor expression^31^. Our results demonstrated that treatment results in the downregulation of these genes, which might be led to swarming inhibition.

An optimized antiadhesive compound should interact with the adhesins of the pathogen, leading to significant inhibition of the docking process between bacteria and eukaryotic cells^32^. As a result, AME reduces the adhesion of *P. mirabilis* on our constructed bladder phantom model and consequently affects the calcium deposition in the catheter.

There are limited data about the molecular investigation for the ability of AME in the prevention of UTI_s_. As a result, AME, a widely used medicinal plant in folk medicine, might strongly regulate QS and biofilm formation of *P. mirabilis* and could decrease the amount of calcium deposited on the catheter. Moreover, the concentrations used do not show cytotoxicity suggests that this extract has the potential to be considered for further studies on the topics, including the prevention of UTIs caused by *P. mirabilis*. This study showed that AME as a natural compound reduced biofilm formation of *P. mirabilis* by targeting virulence factor genes, quorum sensing, and other strategies that include preventing the adhesion of *P. mirabilis* to the cells. The results suggest that *A. maurorum* extract might have the potential to be considered for preventing UTIs caused by *P. mirabilis*.

## Materials and methods

### Preparation of *Alhagi* crude extract

The whole part of *the A. maurorum* plant was collected during the flowering stage in July 2020 from the desert areas around Isfahan province (Gaz, Isfahan, Iran). The plant samples were authenticated by a specialist. The material was identified by J.B and M.G. A voucher specimen of the material is retained in the archives of the Department of Pharmacognosy, Isfahan Pharmaceutical Sciences Research Center under the designation 38,330 (FUMH). Ten grams of freshly powdered plant material were extracted with 100 mL of 50% ethanol for 15 min (3 × 5 min) under ice-cooling by rotor–stator extractor (Ultraturrax®) at maximum rotor speed. The extraction step was repeated 3 times. Then, the suspension was centrifuged at 5.000 × g for 15 min, and the clear supernatant was dried by a rotary vacuum evaporator to yield 2.0 g of dry extract (herbal material: extract ratio = 5:1). The *A. maurorum* extract (AME) was stored at − 20 °C in sealed containers under a vacuum^12^.

### Essential oil (EO) isolation

The powdered *A. maurorum* (100 g) was subjected to hydro distillation for 4 h using the Clevenger apparatus (Clevenger, 1928). Then, the EOs were dehydrated by olive oil and stored in tightly sealed glass vials at − 20 °C for further analysis.

### Gas chromatography-mass spectrometry (GC–MS) analysis

The gas chromatograph was equipped with a programmable split/spitless injector, a capillary column, and a programmable oven. A sample volume of 2 μL was injected at 271 °C, in spitless mode, in a baffle Siltek-deactivated liner (2 mm × 2.75 mm × 120 mm) provided by Thermo Fisher Scientific. Samples were analyzed via gas chromatography (Agilent USB-393752) equipped with an FID detector and capillary column^33^.

### High-performance liquid chromatographic (HPLC)

Active phytochemical compounds were determined in the aqueous extract of the leaves by HPLC. A 100 mg of dried extract was hydrolyzed in HCl: Tetrahydrofuran (2.5 M) for 1 h. Flavonoid analytes were extracted into a water-soluble solvent (HCL (2 N) and diethyl ether), followed by partitioning of the analyte molecules in an organic solvent in the presence of a salt mixture (salting-out effect). The binary mobile phase consisted of solvent A (water: H_3_PO_4_ 10 mM; 99:1; v/v) and solvent B (acetonitrile). NUCLEOSIL® 100–5 RP-18 (Thermo scientific column, 150 mm × 4.6 mm) was used to separate phenolic compounds with isocratic elution: 75% A to 25% B at a flow rate of 1.2 ml/min, the time rum was over 10 min. A UV detector detected the phenolic acids and flavonoids at 200–500 nm wavelength. A standard calibration curve in the range of 0.005 to 0.1 mg/ml was prepared for quantitative analysis using different concentrations of standards (0.005, 0.025, 0.01, 0.1 mg/ml). The chromatographic peaks were identified by comparing the retention time of analytics with that of the reference compounds. The relationship between the concentration and peak area of the standard was measured using the minimum square method (R^2^ value).

### Determination of cell viability (MTT assay)

Cytotoxic assays were done in the HeLa cell line (ATCC CCL-2) obtained from the National Cell Bank of Iran, Pasteur Institute of Iran (Tehran, I.R. Iran). HeLa cells (0.5 × 10^4^ cells/ well) were seeded in 96 well-microtiter plates in the presence of Dulbecco’s Modified Eagle Medium (DMEM, Gibco, USA), supplemented with 5% FBS (Gibco, USA), and incubated for 12 h in a humidified atmosphere with 5% CO_2_ at 37 °C. AME was solubilized in water to give a stock solution with a 2 mg/mL final concentration. Serial ten-fold dilutions of AME in DMEM were prepared to reach 62.5–1000 μg/mL concentrations. Then, 100 µl of each dilution was added to each well. Hela cells with the growth medium were used as control. After incubation for 24 h, the viability of the cells were assessed by MTT assay as described previously^34^.

### Microbial isolation and identification

In this study, 40 *P. mirabilis* were isolated from the catheters collected from intensive care unit (ICU) patients of various hospitals in Isfahan and confirmed with conventional biochemical and genetic tests^35^. The *P. mirabilis* ATCC7002 was used as a standard control.

### Antimicrobial activity of AME, growth measurement and MIC of AME and the bioactive compounds of AME (quercetin, tamarixetin, and trans-anethole)

The antimicrobial activity of AME was initially tested against *P. mirabilis* strain (ATCC7002) by the agar well diffusion method^36^. A freshly prepared culture of *P. mirabilis* was adjusted to OD_620_ of 0.2 and suspended in sterile PBS. 100 μL of the bacterial suspension were swabbed on the Muller-Hinton plate and spread homogeneously. Then, 6 mm of the wells were cut into the agar plate, followed by adding 50 µL of AME dissolved in PBS at different concentrations (62.5–2000 μg/mL). Plates were incubated at 37℃ for 24 h. The inhibition zones around the tested wells were measured to detect the AME range of effect against *P. mirabilis*. The disc antibiotic model of ofloxacin (5 μg/mL) was put on an agar surface as a positive control, and PBS was added in well served as a negative control.

For growth measurement, the overnight culture of *P. mirabilis* 7002 (10^8^ CFU/ml) was inoculated into 10 mL of Luria Bertani Broth, and the OD_620_ value was adjusted to 0.1. Then, 50 µL of the culture was transferred into each well of a 96-well polystyrene microtiter plate that contained 100 µL of LB broth. Subsequently, AME at different final concentrations (125–1000 μg/mL) was added to the wells, and the cultures were incubated at 37 °C for 24 h while shaking (180 rpm). Gentamicin (100 μg/mL) and liquid medium served as positive and negative controls, respectively. The bacterial growth was monitored at 30 min intervals, and the OD_620_ nm was recorded by a microplate reader (Infinite F50, Tecan)^37^. The test was done in triplicate for each concentration. MIC values of crude extract and its essential oils were determined using the microdilution broth method described by Wiegand et al.^28^.

### Adhesion assay

HeLa cells (0.5 × 10^5^ cells/ well) were seeded in 24-well plates with/without different extract concentrations (125–1000 μg/mL) and infected with 10^6^ CFU/mL of *P. mirabilis* and incubated at 37 °C under 5% CO_2_ for two hours. The wells were washed three times with PBS to remove non-adherent bacteria. To detect adherent bacteria, cell cultures were treated with 500 μl 0.025% Triton X-100 for 5 min at 37 °C in 5% CO2 to detach and lyse the cell monolayer. After that, the cell lysates were diluted in ten serial dilutions. Bacterial colonies were counted after the cell lysates were inoculated on Trypticase Soy Agar (TSA) and incubated at 37 °C for 24 h. The number of bacterial colonies in treated plates was compared to the control^29^.

### Swarming motility assay on agar

Fifty microliters of the series of AME (125–1000 μg/mL), quercetin (1 mg/mL), tamarixetin (1 mg/mL), trans-anethole (1 mg/mL) were mixed with 10 ml of molten Mueller–Hinton agar medium and poured immediately over the surface of the plate as an overlay. The plate was point-inoculated with an overnight culture of *P. mirabilis* (ATCC7002) once the overlaid agar had solidified and incubated at 37 °C for 3 days. The extent of swarming was determined by measuring the area of the colony^30^. The test was done in triplicate for each concentration.

### Static biofilm assay

In this study, twelve MDR *P. mirabilis* strains were isolated from CAUTIs of patients attending reference AL-Zahra hospital (Isfahan, Iran) and identified, as described previously^35^. The *P. mirabilis* clinical isolates were assessed for their biofilm activity in a microtiter plate according to the previously described method^35^. The clinical isolates which had strong biofilm formation were chosen for further investigation. To study the extract's antibiofilm activity, 100 µl (OD_620_ = 0.1) of each isolate culture were plated into a 96-well polystyrene microtiter plate and incubated for 72 h at 37 °C. Then, the media were discarded, and the biofilms were washed with PBS (pH 7.2). The biofilms were supplemented with 100 µl of the AME (125–1000 μg/mL), quercetin (62.5–1000 μg/mL), tamarixetin (62.5 μg/mL–1 mg/mL), trans-anethole (62.5 μg/mL–2 mg/mL), individually and incubated for 18 h at 37 °C. Then, the media were removed, and the wells were fixed with 96% ethanol, followed by staining with 0.1% crystal violet for 15 min. The wells were consequently washed 5 times with H_2_O, solubilized in acetone 33% and ethanol 80% (1:1). The amount of biomass was quantified by measuring the OD_620_ using an ELISA- microtiter plate reader (Infinite F50, Tecan). Each treatment was done in triplicates. As a control, 100 µl of nutrient broth was added to the original biofilm of the isolated *P. mirabilis*. The percentage of biofilm reduction is calculated with this formula: (control untreated OD_590_ nm—the mean of three replicants test OD_590_ nm/control untreated OD_590_ nm) × 100^38^. All of the OD of tests were normalized by subtracting the OD_590_ of stained treated and untreated (bacteria only) from the OD_590_ of stained control wells containing bacteria-free medium only.

### Qualitative screening of anti-QS activity

We used pigmented biosensor strain of *Janthinobacterium lividum* (ATCC 12472) as a reporter to study the anti-QS potential of the four crude *A. maurorum* extracts^39^. Agar overlay assay was done using 5 ml of molten soft Luria–Bertani (LB) agar (0.3% agar, 45℃), and 50 μL of the freshly prepared culture of the *J. lividum* (OD_620_ = 0.7) was then added before plating the supernatants on the media. The agar-culture solution was immediately poured over the surface of pre-warmed LB agar plates. Then, 20μL of the AME (125–4000 μg/mL) was pipetted on sterile paper discs and let to dry. The discs were placed on the solidified agar. The plates were incubated overnight at 30 °C. Antibacterial activity was revealed through a zone of clearance at the center, and QS inhibition was observed around a colorless, opaque zone with intact bacteria. DMSO was used as a control^40^. This assay was performed in triplicate.

### Quantitative anti-QS assay

Quantitative evaluation of QS inhibitory activity of the AME was carried out based on their ability to inhibit the production of purple pigment violacein by *J. lividum* ATCC 12472. The strain was cultured aerobically in LB at 30 °C supplemented with the optimal concentrations determined by a qualitative anti-QS test (125–1000 μg/mL). Eugenol (0.625 mg/mL; Sigma, St. Louis, MO, USA) was used as QSI-positive control. One milliliter of an overnight culture of the *J. lividum* was centrifuged (13,000 rpm, 10 min) to precipitate the insoluble violacein, and the pellet was evenly resuspended in 1 mL of DMSO. The solution was centrifuged (13,000 rpm, 10 min) to remove the cells, and the violacein was quantified at OD_620_ nm using a UV spectrophotometer (UV-1800, Shimadzu, Kyoto, Japan). The percentage of violacein inhibition was calculated by the following formula: Percentage of violacein inhibition = (control OD_620_ nm − test OD_620_ nm/control OD_620_ nm) × 100^40^.

### Bladder phantom model for treating the biofilm with crude extract

In vitro bladder models, originally described by Stickler et al.^41^, were set up and operated. Size-18 French all-silicone Foley catheters (Chenkang, China) were used in all experiments and were inserted into the bladder via an outlet before retention balloons were inflated with 10 ml of sterile water. To form a sterile and closed drainage system, catheters were attached to a drainage bag. *P. mirabilis* 7002 suspensions (10^10^ CFU, representing late-stage infection) were inoculated directly into the residual bladder urine, and flow was suspended for 1 h to permit cells to establish within the system. At 45 min after bacterial inoculation, test models were treated with an optimal concentration of AME in a volume of 1 ml; the flow was restored 15 min later. The amount of deposited calcium on the primary 2 cm of the catheter was measured and compared with the control. pH was also measured at the start and end of experiments by sampling the medium in the bladder^42^.

### Quantification of crystalline biofilm formation on catheter sections

To measure the levels of crystalline biofilm formation and catheter encrustation in control and extract-treated models, the amount of calcium present on catheter sections removed from bladder models run for a set time (18 h) was quantified by flame photometry^42^.

### Quantitative real-time PCR analysis

The quantitative real-time PCR (qRT-PCR) assay was carried out to study the effect of AME on the expression of QS and adhesion genes (*mrpA*, *pmfA*, *luxS*, *rsmA*, and *rsbA*) of *P. mirabilis.* An overnight inoculated pooled urine with *P. mirabilis*7002 was transferred to fresh urine, treated with an optimal concentration of AME, and incubated for different time intervals (4, 16, and 48 h) at 37 °C. Then, cells were washed with sterile PBS (pH 7.2) three times and collected after 10 min centrifugation at 4 °C. Total RNA was extracted from bacterial cells using an RNA extraction kit (Jena Bioscience, Germany) following the manufacturer’s instructions. Reverse transcription PCR was conducted, and cDNA was synthesized according to the Jena bioscience kit (Germany) protocol. A qRT-PCR was performed on an ABI system (Applied Biosystems StepOne PlusTM, USA). Each 20 µL reaction contained 2 × Master Mix (SYBR® Green Ampliqon, Denmark), diluted cDNA (5 ng/μL), primers (10 pM of each forward and reverse primers), and RNase-free ddH_2_O. The thermocycling conditions were as follows: denaturation for 10 min at 95 °C, followed by 40 cycles of denaturation for 15 s at 95 °C, annealing, and extension at 54 °C for 60 s. 16 s rRNA was used as an internal control. The primers used in this study were designed by the online tool Primer 3 web version 4.0.0 and listed in Table 2. All samples were run in triplicate. The relative expression of target genes was calculated by the conventional 2^−ΔΔCT^ method^43^.

**Table 2:** The list of primers sequences for real-time PCR.

Gene	Primer sequence (5′–3′)	Product (bp)	References
*mrpA*	F: TGCTGCATTAAAAGATGGTGGC R: TTTGTTTACCACCCGCATCG	200	This study
*pmfA*	F: GGCTGCGGCTTTAGTATTTG R: GGCTTGAAGATGCTGCTAATC	146	This study
*luxS*	F: AAAGCCATGCCTGAGAAAGG R: CGACATCCCATTGGCGAAATA	116	This study
*rsmA*	F: AGCCTTTAATCAGCGCCGTA R: GCGTGTTGCTGTTGTGATGA	165	This study
*rsbA*	F: CGCTATCACGCTAACCAACTA R: GCGTCCTTCAAGCCAATAAAC	120	This study
*16SrRNA*	F: ATGTTGGGTTAAGTCCCG R: CTAGCGATTCCRRCTTCA	256	^44^

### Statistical analysis

Statistical analysis was performed by the SPSS software package (Version v16, IBM Corporation, Armonk, NY, USA). All results were presented as mean ± standard deviation (SD). One-way ANOVA plus post-hoc Tukey test or two-tailed paired *t*-test was used to evaluate statistical significance between samples. Statistical significance was regarded as *p* values < 0.05.

### Ethics approval and collection permission

Ethics approval was obtained from the Ethics Committee of Isfahan University of Medical Sciences (IR. MUI. MED.REC.1398.259). All methods were performed in accordance with the relevant guidelines and regulations.

## Data Availability

All data generated or analyzed during this study are included in this published article.
